# Detection of associations with rare and common SNPs for quantitative traits: a nonparametric Bayes-based approach

**DOI:** 10.1186/1753-6561-5-S9-S10

**Published:** 2011-11-29

**Authors:** Lili Ding, Tesfaye M Baye, Hua He, Xue Zhang, Brad G Kurowski, Lisa J Martin

**Affiliations:** 1 Division of Biostatistics and Epidemiology, Cincinnati Children’s Hospital Medical Center, 3333 Burnet Avenue, Cincinnati, OH 45229, USA; 2 Department of Pediatrics, University of Cincinnati College of Medicine, Cincinnati OH 45229, USA; 3 Division of Asthma Research, Cincinnati Children’s Hospital Medical Center, 3333 Burnet Avenue, Cincinnati, OH 45229, USA; 4 Division of Human Genetics, Cincinnati Children’s Hospital Medical Center, 3333 Burnet Avenue, Cincinnati, OH 45229, USA; 5 Division of Physical Medicine and Rehabilitation, Cincinnati Children’s Hospital Medical Center, 3333 Burnet Avenue, Cincinnati, OH 45229, USA

## Abstract

We propose a nonparametric Bayes-based clustering algorithm to detect associations with rare and common single-nucleotide polymorphisms (SNPs) for quantitative traits. Unlike current methods, our approach identifies associations with rare genetic variants at the variant level, not the gene level. In this method, we use a Dirichlet process prior for the distribution of SNP-specific regression coefficients, conduct hierarchical clustering with a distance measure derived from posterior pairwise probabilities of two SNPs having the same regression coefficient, and explore data-driven approaches to select the number of clusters. SNPs falling inside the largest cluster have relatively low or close to zero estimates of regression coefficients and are considered not associated with the trait. SNPs falling outside the largest cluster have relatively high estimates of regression coefficients and are considered potential risk variants. Using the data from the Genetic Analysis Workshop 17, we successfully detected associations with both rare and common SNPs for a quantitative trait. We conclude that our method provides a novel and broadly applicable strategy for obtaining association results with a reasonably low proportion of false discovery and that it can be routinely used in resequencing studies.

## Background

The two highly debated hypotheses on the genetic basis of complex human diseases are the common disease/common variant (CDCV) hypothesis and the common disease/rare variant (CDRV) hypothesis [1]. The CDCV hypothesis states that common diseases are caused by common variants (minor allele frequencies [MAF] > 5%) with small to modest effects. The CDRV hypothesis, on the other hand, argues that common diseases are caused by multiple rare variants (MAF < 5%), each with moderate to high penetrance. Although both common and rare variants likely play a role in complex human diseases, most statistical strategies for association analysis have been developed under the CDCV assumption, except recent work by Li and Leal [2] and Han and Pan [3]. A key strategy for association analysis with rare variants is to study the cumulative effect of multiple rare variants within the same gene or linkage disequilibrium block [2,4,5]. However, these methods identify genetic risk factors at the gene level, not the variant level. We propose a nonparametric Bayes-based approach to detect associations with both rare and common genetic variants for quantitative traits. This approach clusters single-nucleotide polymorphisms (SNPs) according to the magnitude of SNP-specific regression coefficients. SNPs clustered together could come from different linkage disequilibrium blocks, genes, or even different chromosomes and could have quite different MAFs.

## Methods

Suppose that for each individual *i* (*i* = 1, 2, …, *n*) we observe *y_i_*, a quantitative trait; *z_i_*, a *p*-dimensional vector of individual-specific covariates, such as age and sex; and *x_i_* = (*x_i_*_1_, *x_i_*_2_, …, *x_iJ_*) , genotypes at *J* SNPs. Here, we assume an additive genetic model; thus *x_ij_* = 0, 1, or 2, representing the number of minor alleles present at SNP *j* of individual *i*. A regression model on the quantitative trait is given by:(1)

for *i* = 1, 2, …, *n*, where *γ* is a vector of regression coefficients, including the intercept and slopes for individual-specific covariates, the *β_j_* are the SNP-specific regression coefficients, and *ε_i_* is the error term. We specify the following prior distributions for the model parameters: , *β_j_* ~ *G*, *G* ~ DP(*α*, *G*_0_), and *σ* ~ *U*(*a*, *b*). Here, *v*^2^ and *b* >*a* ≥ 0 are prespecified hyperparameters,  is a (*p* + 1)-dimensional normal distribution with mean vector **μ** and variance-covariance matrix Σ, **0** is a vector of zeros, **I** is an identity matrix, *G* denotes a random distribution, *U*(*a*, *b*) denotes a uniform distribution between *a* and *b*, and DP(*α*, *G*_0_) is the Dirichlet process.

### Dirichlet process

The Dirichlet process [6] is a probability model on a space of probability distributions. It has two parameters: the base probability distribution *G*_0_ and the precision parameter *α* (>0). If *G* ~ DP(*α*, *G*_0_), then *G*_0_ is the prior expectation of *G* and *α* controls the variance of *G*. Here, we take , which is a normal density truncated below at 0, and use *U*(*c*, *d*), *d* >*c* > 0, as the prior distribution for the precision parameter *α*.

Sethuraman [7] provided a stick-breaking construction of the Dirichlet process, which states that if we have:(2)(3)(4)

and(5)

then(6)

is a random probability distribution generated from DP(*α*, *G*_0_), where *δ_ϕ_k__* denotes a point mass at *ϕ_k_*. It is clear that *G* is discrete with probability 1. Because of the discreteness, the *β_j_* can take on the same value. That is why the Dirichlet process can be used for clustering analysis.

Ishwaran and James [8] studied a truncated version of the Dirichlet process by choosing a truncation level *N* and setting *V_N_* = 1 in the stick-breaking construction. They used the truncated Dirichlet process to approximate Dirichlet process prior distributions and developed a block Gibbs sampling method for Dirichlet process models.

### Clustering

Each iteration of the Gibbs sampler gives a clustering structure of SNP-specific regression coefficients such that coefficients taking the same value are clustered together. The number of clusters and the cluster membership of the coefficients vary across iterations, giving a random sample of clustering structures. Pairwise probabilities of two coefficients being equal are calculated from the posterior samples [9]. A distance measure is derived as 1 minus these pairwise probabilities and is then used in complete linkage hierarchical clustering to obtain a final clustering structure of the SNPs. We study a range of the number of clusters, from as small as 2–5 clusters to as large as 100 clusters. Optimal cluster numbers are also obtained by striking a balance between sensitivity and specificity. In all cases, SNPs in the largest cluster have relatively low or close to zero estimates of regression coefficients and are considered not associated with the trait. SNPs falling outside the largest cluster have relatively high estimates of regression coefficients and are considered potential risk variants. The proportion of false discovery (FDP), defined as the ratio of the number of false discoveries to the total number of discoveries, is examined.

### Application of the method

We illustrate our methods using the data from Genetic Analysis Workshop 17. The analyses were performed with the knowledge of the underlying simulation model [10]. We studied the first 10 replicates of the quantitative trait Q1. Each replicate contains 697 unrelated individuals from 7 populations. To control for population stratification, we conducted principal components analysis on nonsynonymous common SNPs (*n* = 1,379) and included the resulting first two components as covariates, in addition to Age and Smoke. We built our model with 244 nonsynonymous SNPs selected from the vascular endothelial growth factor (VEGF) pathway [11]. These SNPs include all 39 functional SNPs for Q1, of which 23 are private variants (found in one individual, MAF = 0.000717) and 2 are common SNPs. The model was fitted using WinBUGS [12] with *v*^2^ = 1,000, *a* = 0, *b* = 100, *c* = 0.5, *d* = 20, *µ*_0_ = 0.5, and . The truncation level for the Dirichlet process was fixed at 50. For each replicate, 10,000 Markov chain Monte Carlo posterior samples were generated after a burn-in period of 2,000 iterations.

We evaluated our results using two thresholds. When the number of clusters was small (2–5), we defined true positives as true associations identified in at least 2 of the 10 replicates. This threshold was selected to balance the reduced power resulting from small cluster numbers. Indeed, requiring at least two replications for each identified association yielded a reasonably low FDP. When we used the optimal cluster numbers, we defined true positives as true associations detected in no less than eight replicates. We carried out sensitivity analyses on the prior specification for the SNP-specific regression coefficients with *µ*_0_ ranging from 0.1 to 0.5 and  ranging from 0.5 to 2. Similar results were obtained.

## Results and discussion

### Successful identification of associations

Table 1 lists the true discoveries with their MAFs, regression coefficients (*β*) used in the simulation, and frequency of detection. When we used two clusters, we found eight true positives with no false positives. Compared with false negatives, the true positives have either relatively high MAFs or relatively high effect sizes. With 3 clusters, we had 11 true positives and no false positives. With 4 or 5 clusters, we detected 12 and 13 associations, respectively. However, there was one false positive (data not shown) in both cases (FDP ≈ 8%). We also conducted single-SNP-based tests with Bonferroni correction for multiple comparisons. With the criteria that *p* ≤ 0.05/244 in at least 2 of the 10 replicates, 20 associations were detected and 10 of them were true discoveries, giving an FDP of 50%. Compared with single-SNP tests, our method gave a much lower FDP.

**Table 1:** True discoveries in at least two replicates with 2 to 5 clusters

Gene	SNP	MAF	*β*	F2	F3	F4	F5
*FLT1*	C13S523	0.066714	0.64997	**10**	**10**	**10**	**10**
*FLT1*	C13S431	0.017217	0.74136	**9**	**9**	**9**	**9**
*FLT1*	C13S522	0.027977	0.61830	**8**	**8**	**8**	**8**
*VEGFA*	C6S2981	0.002152	1.20645	**6**	**6**	**6**	**7**
*ARNT*	C1S6533	0.011478	0.5619	**5**	**6**	**7**	**7**
*FLT1*	C13S524	0.004304	0.62223	**4**	**4**	**5**	**5**
*KDR*	C4S1884	0.020803	0.29558	**4**	**4**	**4**	**5**
*KDR*	C4S1878	0.164993	0.13573	**2**	**4**	**4**	**4**
*KDR*	C4S1877	0.000717	1.07706	1	**4**	**5**	**6**
*KDR*	C4S1889	0.000717	0.94133	1	**2**	**3**	**5**
*ARNT*	C1S6542	0.002152	0.46026	1	**2**	**2**	**2**
*KDR*	C4S1861	0.002152	0.56311	1	1	1	**2**

F2, F3, F4, F5: frequency of detection (in bold when ≥ 2) over the 10 replicates with 2 to 5 clusters.

### Selection of optimal number of clusters

**Figure 1:**
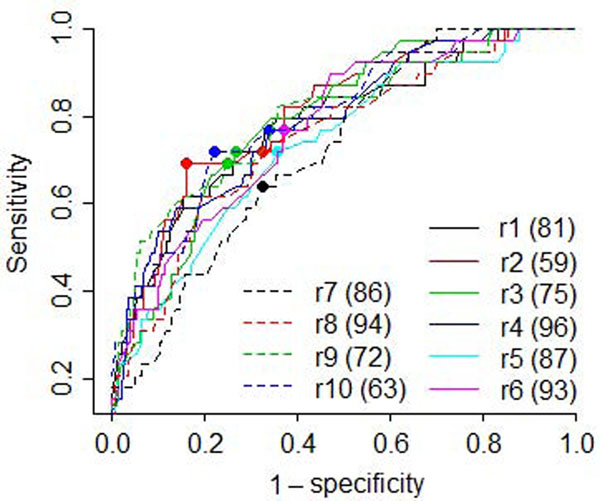
**ROC curves and optimal cluster numbers.** r_1_–r_10_ represent the first 10 replicates of the quantitative trait Q1. Numbers in parentheses and dots on the curves indicate optimal number of clusters for each replicate, which ranges from 59 to 96, with an average of 81.

As the number of clusters increases, more associations may be detected; however, the number of false positives may also increase. To strike a balance between sensitivity and specificity, we examined receiver operating characteristic (ROC) curves (Figure 1) for each replicate. The optimal cluster numbers ranged from 59 to 96, with an average of 81. At the optimal cluster number for each replicate, the average sensitivity and specificity were 0.71 and 0.72, respectively. We then examined the associations detected in these rounds. We had 100% power (detected in all 10 replicates) to detect 10 true associations (Table 2) with 2 false positives (FDP = 17%). Using a threshold of 90% power, five additional true associations were detected and are in the *F* = 9 rows in Table 2, with no additional false positives (FDP = 12%). Using a threshold of 80% power, we detected another four true associations (19 total); however, the number of false positives went up to four (FDP = 17%).

**Table 2:** True discoveries in at least eight replicates with optimal cluster numbers

Gene	SNP	MAF	*β*
***F* = 10, TP = 10, FP = 2, FDP = 17%**			
*ARNT*	C1S6561	0.000717	0.65721
*KDR*	C4S1877	0.000717	1.07706
*KDR*	C4S1879	0.000717	0.61830
*KDR*	C4S1889	0.000717	0.94133
*VEGFC*	C4S4935	0.000717	1.35726
*VEGFA*	C6S2981	0.002152	1.20645
*FLT1*	C13S431	0.017217	0.74136
*FLT1*	C13S522	0.027977	0.61830
*FLT1*	C13S523	0.066714	0.64997
*FLT1*	C13S524	0.004304	0.62223
***F* = 9, TP = 15, FP = 2, FDP = 12%**			
*ELAVL4*	C1S3181	0.000717	0.76911
*ELAVL4*	C1S3182	0.000717	0.30432
*ARNT*	C1S6533	0.011478	0.56190
*FLT1*	C13S399	0.000717	0.39602
*FLT1*	C13S479	0.000717	0.75946
***F* = 8, TP = 19, FP = 4, FDP = 17%**			
*KDR*	C4S1873	0.000717	0.58301
*KDR*	C4S1884	0.020803	0.29558
*FLT4*	C5S5156	0.000717	0.43010
*FLT1*	C13S505	0.000717	0.44850

The items in the bolded rows are the frequency of detection over the 10 replicates (F), the number of true positives (TP), the number of false positives (FP), and the proportion of false discovery (FDP) in each case.

We then evaluated the performance of this method using a specified number of clusters, ranging from 50 to 100. Using only associations identified with 100% power, we had 8 to 10 true positives and at most 2 false negatives (FDP ranging from 10% to 18%). For 90% power, we had 8 to 16 true positives and at most 4 false negatives (FDP ranging from 8% to 20%). Thus cluster numbers of 50 to 100 seem reasonable.

### Characteristics of the true positives and false negatives

**Figure 2:**
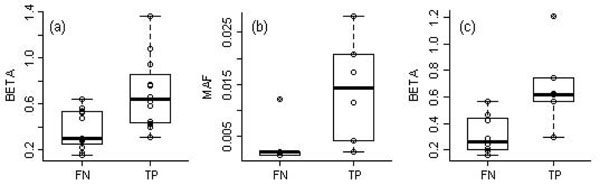
**Boxplot of β and MAF by false-negative or true-positive status.** (a) *β* of private functional SNPs (MAF = 0.000717) for Q1; (b) MAF and (c) *β* of rare but nonprivate functional SNPs (0.000717 < MAF < 0.05) for Q1. FN, false negative; TP, true positive.

Using optimal cluster numbers and the threshold of true positives, we identified 12 of the 23 true associations with private SNPs. As we expected, true positives had overall higher effect sizes than false negatives (Figure 2a). Among the 14 true associations with rare but nonprivate variants, true positives had relatively high MAFs and *β* compared with false negatives, as shown in Figures 2b and 2c.

## Conclusions

We have demonstrated that a novel nonparametric Bayes-based clustering method can be used to identify associations with SNPs for quantitative traits. Importantly, this method is capable of detecting associations with both rare and common genetic variants. Compared with other methods that deal with rare variants, our methods detect genetic risk factors directly at the SNP level. Compared with single-SNP-based methods, the proposed method is more powerful and reliable. It can detect a relatively larger proportion of true associations independent of the MAF of the variants, and it produces a relatively lower proportion of false discoveries.

## Competing interests

The authors declare that there are no competing interests.

## Authors’ contributions

LD conceived and performed the statistical analysis and wrote the manuscript. LJM contributed to the design of the statistical analysis and the writing of the manuscript. TMB, HH, XZ, and BGK helped with the writing of the manuscript. All authors read and approved the final manuscript.
